# Candida albicans *rvs161*Δ and *rvs167*Δ Endocytosis Mutants Are Defective in Invasion into the Oral Cavity

**DOI:** 10.1128/mBio.02503-19

**Published:** 2019-11-12

**Authors:** Shamoon Naseem, Lois M. Douglas, James B. Konopka

**Affiliations:** a Department of Molecular Genetics and Microbiology, Stony Brook University, Stony Brook, New York, USA; Duke University Medical Center

**Keywords:** *Candida albicans*, candidiasis, endocytosis, fungal, invasive growth, oropharyngeal, pathogenesis

## Abstract

Invasive growth in tissues by the human fungal pathogen Candida albicans is promoted by a switch from budding to hyphal morphogenesis that is stimulated by multiple environmental factors that can vary at different sites of infection. To identify genes that promote invasive growth in the oral cavity to cause oropharyngeal candidiasis (OPC), we first identified C. albicans mutants that failed to invade agar medium. Analysis of nine severely defective mutants in a mouse model of OPC revealed that the strongest defects were seen for the *rvs161*Δ and *rvs167*Δ mutants, which lack amphiphysin proteins needed for endocytosis. The *rvs*Δ mutants initially adhered to the tongue but failed to invade efficiently and were lost from the oral cavity. Previous studies indicated that *rvs*Δ mutants formed filamentous hyphae in the kidney albeit with morphological abnormalities, suggesting that the *rvs*Δ mutants were influenced by factors that vary at different sites of infection. Consistent with this, increasing concentrations of CO_2_, an inducer of hyphal growth that is more abundant in internal organs than air, partially rescued the invasive-growth defects of the *rvs*Δ mutants *in vitro*. Interestingly, preinduction of the *rvs*Δ mutants to form hyphae prior to introduction into the oral cavity restored their ability to cause OPC, identifying a key role for endocytosis in initiating invasive hyphal growth. These results highlight the influence of distinct environmental factors in promoting invasive hyphal growth in the oral cavity and indicate that blocking endocytosis could have therapeutic value in preventing the initiation of OPC.

## Abstract (precis)

Oropharyngeal candidiasis (OPC) is a common fungal infection that is associated with severe morbidity. Another concern is that patients at risk for developing OPC often take long courses of antifungal drugs, which can lead to the emergence of drug-resistant C. albicans strains. We therefore identified nine mutants with defects in undergoing invasive hyphal growth in the oral cavity, increasing the number of genes known to be involved in OPC by more than 30%. The two strongest mutants, *rvs161*Δ and *rvs167*Δ, have defects in endocytosis. The *rvs*Δ mutants appear to have a specific defect in initiating invasive growth, as preinducing the cells to form hyphae prior to infection restored their ability to cause OPC. These results indicate that blocking endocytosis could have therapeutic value in preventing the initiation of OPC without leading to development of resistance against drugs currently used to treat fungal infections.

## INTRODUCTION

The human fungal pathogen Candida albicans is present on skin and mucosa as a harmless commensal. However, when host defense systems are impaired, C. albicans can grow invasively in a wide range of tissues, leading to severe pathological consequences. One of the most common types of infection is oropharyngeal candidiasis (OPC), which is due to invasive growth of C. albicans in the oral mucosa. OPC can result from a number of different underlying risk factors (1, 2). One major susceptibility factor is immunosuppression, especially defects in T cells or the interleukin-17 (IL-17) signal pathway (3, 4). For example, OPC is usually one of the first clinical signs to appear as HIV-infected individuals progress to AIDS (5, 6). Other risk factors include the use of corticosteroid inhalers, the use of chemotherapy for solid cancers, smoking, diabetes, the use of broad-spectrum antibiotics, and denture wear (7). OPC is also commonly seen under conditions that dry out the mouth (xerostomia), which can occur as a side effect of certain medications or as a consequence of some autoimmune diseases (8).

A key step in the development of OPC is for C. albicans to undergo a transition from budding to filamentous hyphal growth (1, 9). Hyphal cells display adhesin proteins on their surface that promote the ability of C. albicans to initially colonize the oral cavity by binding to the epithelial cells lining the oral mucosa (10, 11). Hyphal growth is then thought to mediate two mechanisms that enable C. albicans to breach the epithelial cell barrier in the oral mucosa and initiate OPC (1). One mechanism, known as active penetration, involves hyphae pushing their way into epithelial cells or between adjacent epithelial cells (12, 13). Another mechanism for invasion involves C. albicans cells being endocytosed by epithelial cells, which is stimulated by an interaction between the Als3 and Ssa1 proteins on the hyphal surface with cadherins and growth factor receptors on the surface of epithelial cells, leading to the activation of aryl hydrocarbon receptors (10, 11, 14, 15). The adhesin proteins also increase the attachment of fungal cells to each other to promote biofilm formation on the surface of the oral cavity (9, 16). Invasion is further promoted because stimulation of hyphal morphogenesis coordinately induces genes that promote epithelial cell damage, including proteases and the pore-forming peptide candidalysin (17).

Previous studies have identified genes that regulate the ability of C. albicans to switch from budding to hyphal growth in response to a wide range of different environmental conditions (18–20). However, several factors limit our understanding of invasive growth. One is that many hyphal mutants are defective only in response to weak stimuli and can still be induced by stronger stimuli, such as those encountered in the host (21–23). Another major factor is that many mutants cause a hyphal defect only in liquid medium and are still capable of invasive hyphal growth in a solid substrate. This phenotype is due at least in part to the fact that contact with a solid substrate triggers independent hyphal pathways (22, 24–26). Therefore, to identify novel functions that are important for invasive hyphal growth in OPC, we carried out a genetic screen for C. albicans mutants that were defective in invasion into a solid agar medium under a variety of strongly inducing conditions. Analysis of the strongest mutants that we identified in a mouse model of OPC (27) revealed an important role for endocytosis in the initiation of invasive hyphal growth.

## RESULTS

### Genetic screen for invasive-growth mutants.

Genes required for invasive growth were identified by screening three libraries of mutant C. albicans strains (21, 28, 29), along with mutant strains in our own collection (see Table S1 in the supplemental material). The initial screen examined invasion into agar containing 4% serum at 37°C, which potently induces invasive growth. We identified a large set of candidates with various degrees of defects, so further analysis was restricted to the strongest mutants. We also excluded strains carrying mutations affecting transcription from further analysis (Table S1), as several transcription factor mutants have been studied previously in OPC (30–35). The remaining mutant strains were then tested for their ability to invade agar under other strongly hypha-inducing conditions, including the sugar *N*-acetylglucosamine, alkaline pH (pH 8.5 buffer), and spider medium (mannitol and nutrient broth) (18–20). We also screened mutants using a higher agar concentration (4%), as a more rigid agar matrix promotes a robust invasive response (22).

10.1128/mBio.02503-19.3 TABLE S1 C. albicans strains examined for invasive growth in agar. Download Table S1, XLSX file, 0.2 MB. Copyright © 2019 Naseem et al. 2019 Naseem et al. This content is distributed under the terms of the Creative Commons Attribution 4.0 International license.

**FIG 1:**
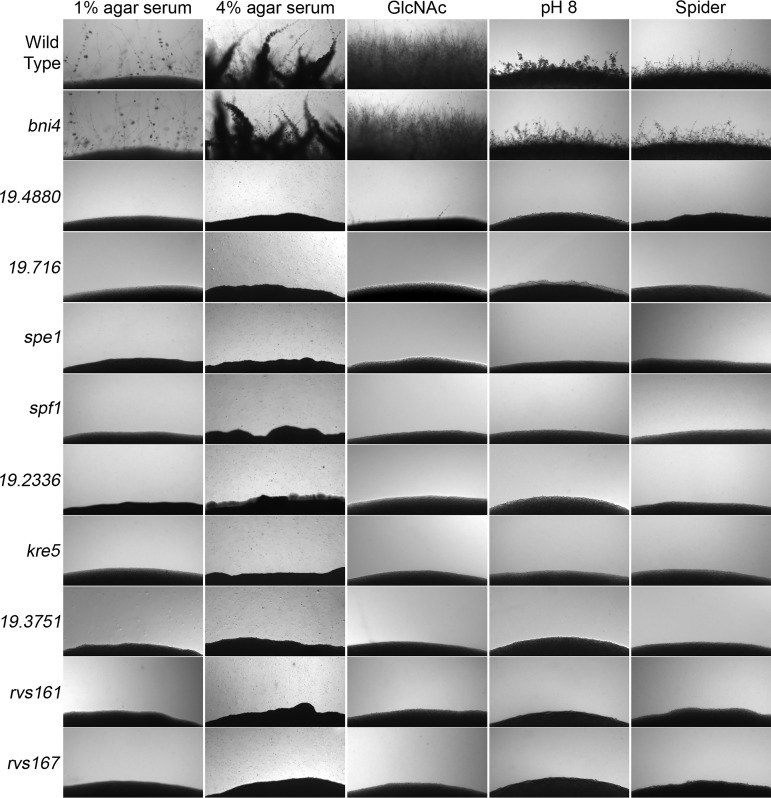
C. albicans mutants defective in growing invasively in agar. The mutants indicated on the left were spotted onto different hypha-inducing agar media, as indicated at the top. The plates were incubated for 2 days at 37°C, and the edge of the spot of cells was then photographed to record the extent of invasion into the agar. The strains are described in Table S2 in the supplemental material.

Nine mutants that failed to invade agar under the different conditions after 2 days at 37°C were identified (Fig. 1). The mutants are defective in diverse functions, including endocytosis (*rvs161*Δ and *rvs167*Δ), calcium homeostasis (*spf1*Δ), decarboxylation of ornithine (*spe1*Δ), protein kinase activity (*orf19.3751*Δ), and 1,6-β-d-glucan biosynthesis (*kre5*Δ). Three other mutants have defects in genes whose function has not yet been defined (*orf19.4880*Δ, *orf19.716*Δ, and *orf19.2336*Δ). Hyphal defects were previously reported for the *spf1*Δ (36), *kre5*Δ (37), *spe1*Δ (38), *rvs161*Δ, and *rvs167*Δ (39) mutants. The predicted gene functions are summarized in Table 1.

**TABLE 1:** Genes analyzed for a role in oral candidiasis

ORF^*a*^	Gene name	Systematic name	Description and/or function
19.4880		C1_10110W	Similar to glycine-rich domain proteins involved in response to stress; not conserved in Saccharomyces cerevisiae
19.716		CR_06500C	Similar to the septicolysin family of bacterial cytolysins; not conserved in S. cerevisiae
19.6032	*SPE1*	C1_00740C	Ornithine decarboxylase
19.30	*SPF1*	C2_06540C	P-type calcium-transporting ATPase, involved in control of calcium homeostasis
19.2336		C1_10810W	Similar to S. cerevisiae *PRY3* and the Pry family pathogenesis-related proteins
19.290	*KRE5*	C3_02960C	Involved in 1,6-β-d-glucan biosynthesis
19.3751		CR_02210W	Putative Ser/Thr protein kinase similar to S. cerevisiae *KIN4* and *FRK1*
19.7124	*RVS161*	C7_00020C	BAR domain protein involved in actin organization and endocytosis
19.1220	*RVS167*	C6_04040C	BAR domain protein involved in actin organization and endocytosis

a ORF, open reading frame.

10.1128/mBio.02503-19.4 TABLE S2 C. albicans strains used in this study. Download Table S2, PDF file, 0.02 MB. Copyright © 2019 Naseem et al. 2019 Naseem et al. This content is distributed under the terms of the Creative Commons Attribution 4.0 International license.

### Mutants with invasion defects *in vitro* show a reduced ability to cause OPC in mice.

**FIG 2:**
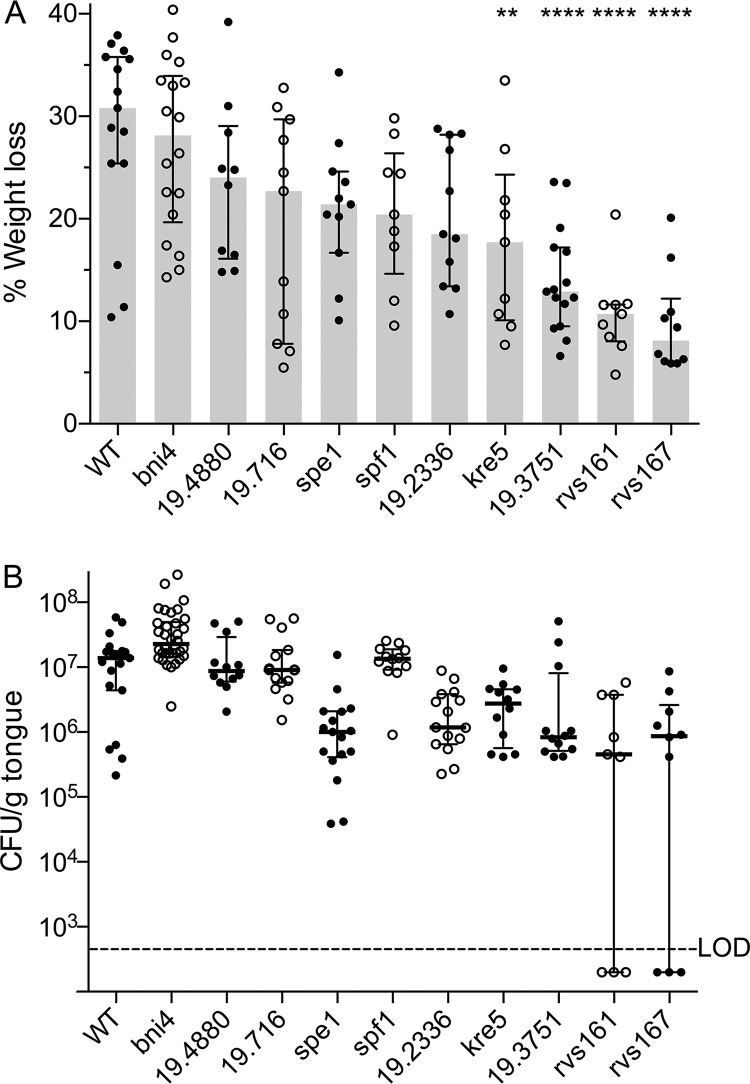
Ability of invasive-growth mutants to cause OPC in mice. (A) Extent of weight loss 4 days after oral infection of C57BL/6 mice with the indicated C. albicans wild-type or mutant strains. The statistical differences between the wild type (WT) and the following mutants were determined by one-way analysis of variance (ANOVA) corrected for multiple comparisons with Tukey’s test: *kre5*Δ (*P* < 0.01), *orf19.3751*Δ, *rvs161*Δ, and *rvs167*Δ (*P* < 0.001). (B) CFU per gram of tongue for the indicated strains after 4 days of infection. The mutants that were significantly different (*P* < 0.001) than the *bni4*Δ control strain by a nonparametric Kruskal-Wallis test corrected for multiple comparisons with Dunn’s test were the *spe1*Δ, *orf19.2336*Δ, *kre5*Δ, *orf19.3751*Δ, *rvs161*Δ, and *rvs167*Δ mutants. The gray bars indicate the medians, and the error bars indicate the upper and lower quartiles. The strains are described in Table S2 in the supplemental material. LOD indicates the limit of detection. Bars show the medians and interquartile ranges. The results represent data from at least three independent infections for each mutant strain. In each set of infections, three mice were infected with a specific mutant strain (see Materials and Methods).

C. albicans wild-type control strains and nine selected mutants were examined in a previously described mouse model of OPC (27). The mice were immunocompromised with cortisone acetate, and a swab containing C. albicans was then placed under the tongue to initiate infection (see Materials and Methods). Four days after infection, the extent of weight loss was determined (Fig. 2A). Weight loss is a good indicator of the severity of OPC, as the morbidity caused by the infection deters mice from eating (27). A prototrophic wild-type control strain, DIC185, and a control mutant strain that was not predicted to be defective in OPC (*bni4*Δ) gave the expected results in that the infected mice lost >20% of their weight after 4 days. The invasion-defective mutants showed various degrees of weight loss (Fig. 2A). However, weight loss was significantly different than the wild-type strain for only the *orf19.716*Δ, *kre5*Δ, *frk1*Δ, *rvs161*Δ, and *rvs167*Δ mutants.

The fungal burden was then determined by plating dilutions of the tongue homogenate to determine the viable CFU (Fig. 2B). All of the mutants showed a trend toward fewer CFU on the tongue. However, the reduced CFU seen for some of the mutants were not statistically significant relative to the wild-type control strain (DIC185), likely due to an assay in which, for unclear reasons, the tongue CFU were low for the wild-type control. In contrast, the CFU of all of the mutants were significantly lower than those of the control mutant strain (*bni4*Δ) (*P* < 0.05). The decrease in CFU generally correlated with weight loss, although there were some exceptions, which are discussed further below. The mutant phenotypes are summarized in Table 2.

**TABLE 2:** Mutant phenotypes

Strain	Time of agar invasion (days)^*a*^	wt loss^*b*^	Fungal burden^*c*^	Hyphae in tongue histology^*d*^	Hyphae in tongue homogenate^*d*^
Wild type	2	++++	++++	++++	++++
*orf19.4880*Δ	4	++++	++++	+++	+++
*orf19.716*Δ	4	++++	++++	++	+
*spe1*Δ	>7	++++	+	−	+/−
*spf1*Δ	4	++++	++++	++	++
*orf19.2336*Δ	4	++++	+++	++	++
*kre5*Δ	>4	++	+++	+	++
*orf19.3751*Δ	>4	++	++++	−	+/−
*rvs161*Δ	ND	+	+/−	−	−
*rvs167*Δ	ND	+	+/−	−	−

a Days of incubation after which the strains started to show detectable invasion into agar containing serum or GlcNAc. > indicates that invasion was seen for only one of the stimuli. Note that none of the mutants showed significant invasive growth in agar medium at alkaline pH or in spider medium after 7 days, whereas the wild-type control showed invasion after 2 days. ND, not determined.

b ++++, not significantly different; ++, about one-half of the weight loss; +, about one-third of the weight loss seen for mice infected with wild-type control C. albicans.

c Measured as median CFU per gram of tongue. ++++, ≤2-fold lower; +++, ≤5-fold lower; +, <8-fold lower; +/−, ∼10-fold lower, with some mice lacking detectable CFU.

d Presence of hyphae. ++++, nearly all hyphae; ++, presence of both hyphae and buds; +, rare hyphae and mostly buds; −, no or rarely detectable hyphae.

The most obvious difference was the strong defect of the *rvs161*Δ and *rvs161*Δ strains. They showed the least weight loss and the lowest CFU. In addition, about 30% of the mice appeared to have no detectable CFU after 4 days of infection by these mutants. The *rvs*Δ strains were the only mutants in which the CFU per gram of tongue were below the limit of detection. There was a good correlation between the amount of whitish fungal growth on the tongue after 4 days (Fig. S1), the extent of weight loss, and CFU data. In particular, the *rvs161*Δ and *rvs167*Δ mutant tongues showed very low levels of fungal growth, in some cases being similar to those of the control tongues from uninfected mice, consistent with some mice infected with the *rvs*Δ mutants having low or no detectable CFU after 4 days.

10.1128/mBio.02503-19.1 FIG S1 Visualization of C. albicans growth on tongues. Growth of C. albicans strains in the oral cavity was documented by photographing tongues after 4 days of infection. Download FIG S1, PDF file, 1.5 MB. Copyright © 2019 Naseem et al. 2019 Naseem et al. This content is distributed under the terms of the Creative Commons Attribution 4.0 International license.

### Histological analysis of invasive hyphal growth in the tongue.

**FIG 3:**
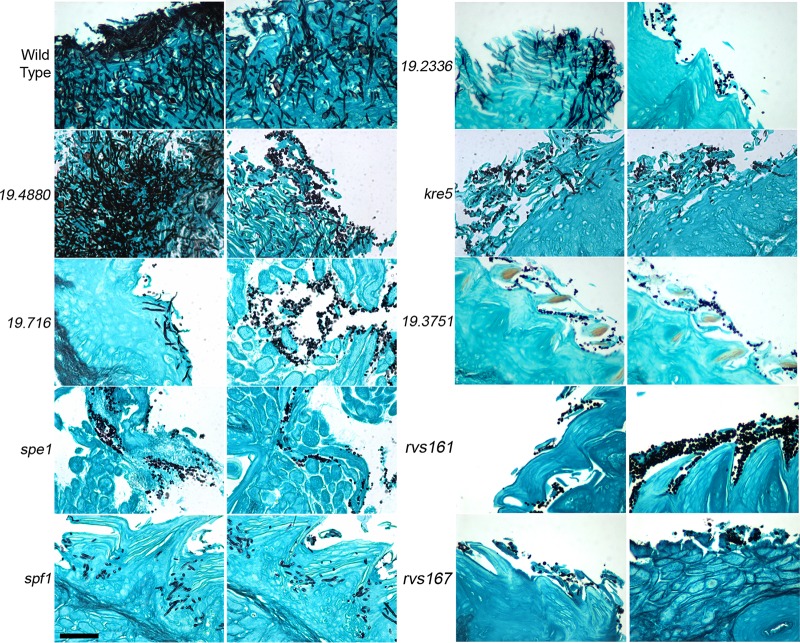
Histological analysis of C. albicans invasive growth in the tongue. Tongues were harvested after 4 days of infection and then processed for histological staining by Grocott’s methenamine silver (GMS). Two representative areas are shown for each mutant. The mutants are ordered according to the extent of weight loss that they caused due to OPC (Fig. 2A). The strains are described in Table S2 in the supplemental material. Bar, 50 μm. Four tongues were analyzed for each mutant.

The morphology of the C. albicans cells and the extent of invasion into the tongue epithelium were examined by histology using Grocott’s methenamine silver (GMS) staining (Fig. 3). This procedure provides strong contrast for sensitive detection of C. albicans in tongue tissue, as the fungal cells are stained black against the blue-green background of the tongue tissue. Two different histological sections are shown for each mutant to illustrate how in some cases the morphology of C. albicans varied in different regions of the tongue. The wild-type control strain always showed extensive invasion of hyphal cells into the epithelium, with few or no budding cells detected. In contrast, all nine mutants showed detectable levels of budding cells. Five of the mutants were also able to form readily detectable invasive hyphal filaments (*orf19.4880*Δ, *orf19.716*Δ, *spf1*Δ, *orf19.2336*Δ, and *kre5*Δ). These mutants were among those that displayed the best ability to cause OPC in mice.

Four of the mutants did not appear to form invasive hyphal filaments in the GMS-stained samples. These mutants were among those that had the weakest ability to cause OPC (*spe1*Δ, *orf19.3751*Δ, *rvs161*Δ, and *rvs167*Δ). The mutant cells appeared to be attached to the periphery of the tongue but were not invasive.

### Analysis of C. albicans morphology in tongue homogenates.

**FIG 4:**
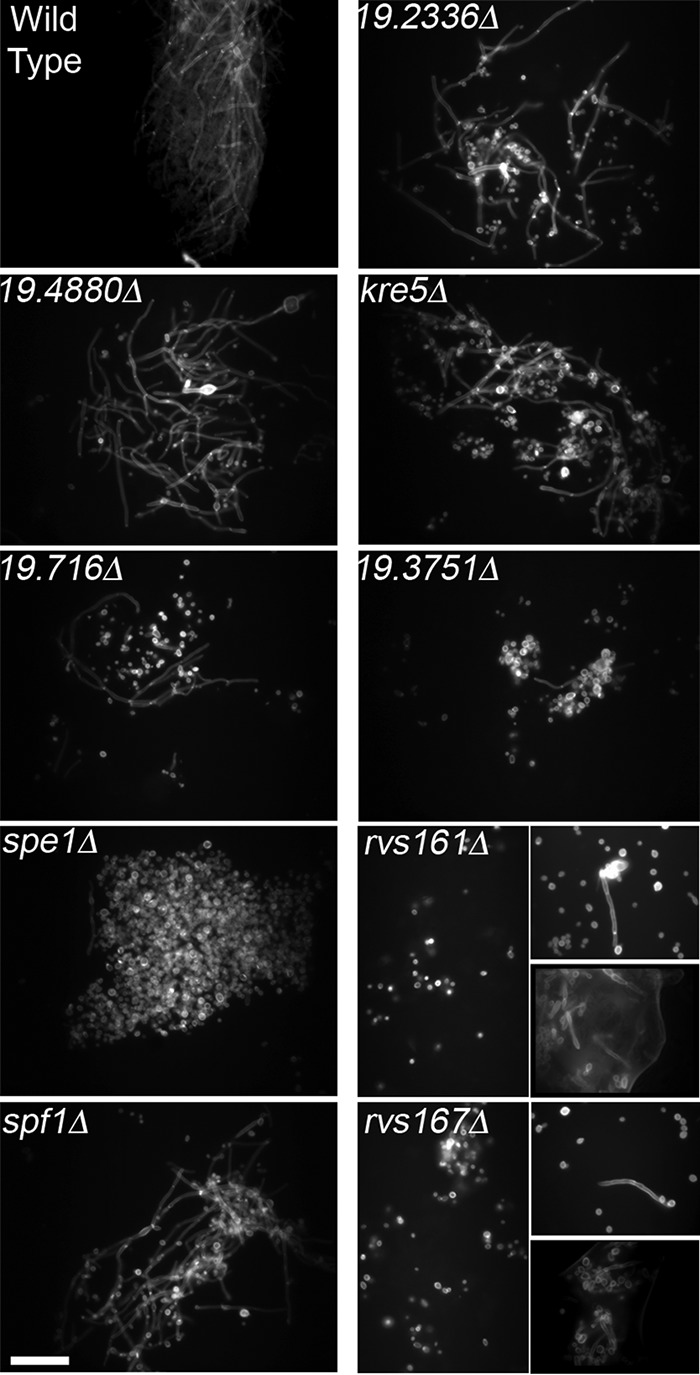
Visualization of C. albicans cell morphology in tongue homogenates. In order to gain a broader representation of C. albicans morphology, infected tongues were homogenized and treated with 1 M KOH to dissolve mouse tissue, and the C. albicans cell walls were then stained with the fluorescent dye calcofluor white and visualized by fluorescence microscopy. The mutants are ordered according to the extent of weight loss that they caused in mice due to OPC (Fig. 2A). The strains are described in Table S2 in the supplemental material. Similar results were obtained in at least two independent infection experiments. Bar, 50 μm.

Although histology is a good way to examine C. albicans invasion, the results represent only a small section of the tongue. To gain a broader representation of C. albicans cell morphology, infected tongues were homogenized and then treated with KOH to dissolve the mouse tissue in a variation of a strategy often used to visualize fungi in clinical samples (40). The C. albicans cell walls survived this treatment and were then stained with the chitin-binding dye calcofluor white to detect cell morphology by fluorescence microscopy (Fig. 4). As expected, essentially all of the wild-type control cells displayed a filamentous hyphal morphology. The mutants that formed invasive hyphal cells detected in GMS-stained tongue sections also showed a significant number of hyphal cells (*orf19.4880*Δ, *orf19.716*Δ, *spf1*Δ, *orf19.2336*Δ, and *kre5*Δ). They were estimated to contain between 10% and 50% hyphal cells, although it was difficult to quantify the exact number of hyphal and budding cells in these samples, in part due to clumping.

The four mutants that did not produce detectable hyphae in the histological samples (*spe1*Δ, *orf19.3751*Δ, *rvs161*Δ, and *rvs167*Δ) were all found to form at least some hyphae in the bulk analysis of the tongue homogenate by calcofluor white staining (Fig. 4). The *spe1*Δ and *orf19.3751*Δ mutants formed hyphae at a very low level that was estimated to be about 1% of the population. The *rvs161*Δ and *rvs167*Δ mutants formed a few detectable hyphal filaments in some infected mice but not others (Fig. 4). This variation correlates with differences in CFU per gram of tongue seen for the *rvs161*Δ and *rvs167*Δ mutants (Fig. 2B) and the strong defect in promoting weight loss in mice (Fig. 2A).

### Time course analysis of OPC in mice infected with the *rvs161*Δ and *rvs167*Δ mutants.

**FIG 5:**
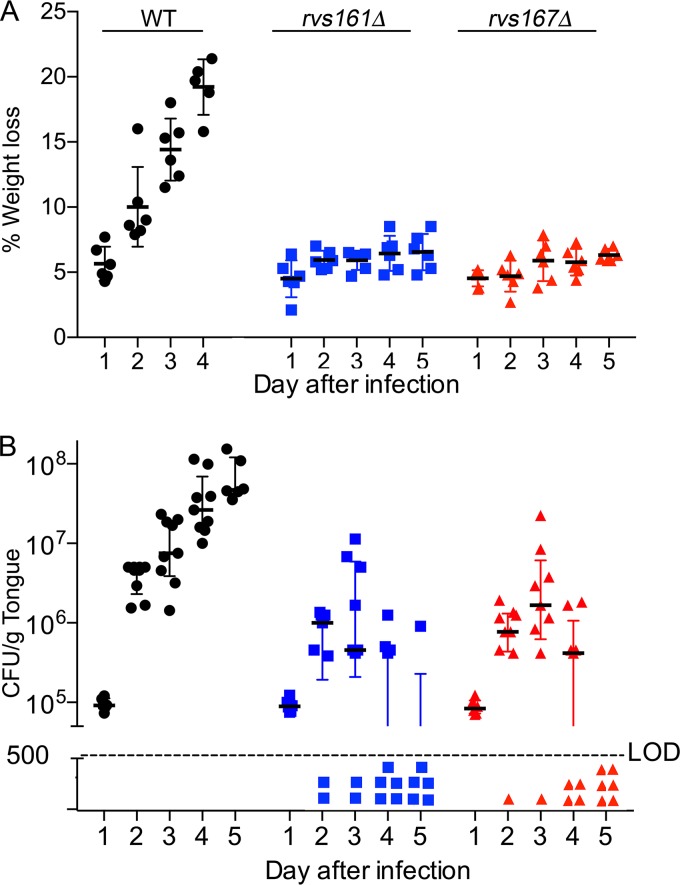
Time course of oral infection by the *rvs161*Δ and *rvs167*Δ mutants. Mice were infected in the oral cavity with C. albicans, and on the indicated day postinfection, the weight of the mouse (A) and the CFU of C. albicans per gram of tongue (B) were then determined. Note that by day 5 postinfection, 5/6 mice infected with the *rvs161*Δ mutant and all 6 mice infected with the *rvs167*Δ mutant lacked detectable CFU on the tongue. LOD indicates the limit of detection. Analysis by one-way ANOVA corrected for multiple comparisons with Tukey’s test indicated that weight loss was significantly different from the wild type at day 2 and beyond for the *rvs161*Δ (*P* < 0.01) and *rvs167*Δ (*P* < 0.001) mutants. The CFU were also significantly different from the wild type for the *rvs161*Δ mutant at days 2 and 3 (*P* < 0.05) and for both *rvs*Δ mutants at days 3 and 4 (*P* < 0.01) using a nonparametric Kruskal-Wallis test corrected for multiple comparisons with Dunn’s test. Results represent the averages of data from two to three independent infections for each time point.

A time course analysis showed that mice infected with wild-type control C. albicans displayed progressive weight loss each day and were euthanized after day 4 due to the extent of weight loss (Fig. 5). In contrast, mice infected with the *rvs161*Δ and *rvs167*Δ mutants lost about 5% of their weight after 1 day of infection, perhaps due to cortisone treatment, but did not show any further significant weight loss. The tongue CFU for the wild type and the mutants were similar at 1 day postinfection (∼10^5^ CFU/g tongue), indicating similar abilities to adhere. However, 2 days after infection, the mutant CFU lagged behind the wild-type CFU. Whereas the wild-type CFU increased about 50-fold on day 2 and proceeded to increase about 2-fold each day after that, the median CFU for the *rvs*Δ mutants increased by only about 10-fold on day 2 and essentially plateaued after that. In addition, by day 2 postinfection, some of the mice infected with the *rvs*Δ mutants no longer had detectable CFU. After 5 days of infection, 5 out of 6 mice infected with the *rvs161*Δ mutant and all 6 mice infected with the *rvs167*Δ mutant lacked detectable CFU. Thus, the *rvs*Δ mutants initially adhered to the tongue, but they were not able to maintain the infection. This correlates with the decreased invasive hyphal filaments for these mutants.

Control studies showed that the strong phenotypes exhibited by the *rvs161*Δ and *rvs167*Δ mutants were rescued in complemented strains in which a wild-type copy of the deleted gene was reintroduced. The complemented strains were similar to the wild-type control strain in their abilities to cause weight loss, CFU per gram of tongue following infection, and invasion of the tongue as judged by histology (Fig. S2).

10.1128/mBio.02503-19.2 FIG S2 Complementation of the *rvs161*Δ and *rvs167*Δ mutant phenotypes by reintroduction of the wild-type genes. The wild-type control strain (DIC185), the *rvs161*Δ complemented strain (*rvs161*Δ + *RVS161*), and the *rvs167*Δ complemented strain (*rvs167*Δ + *RVS167*) were used to infect the oral cavities of mice. There were no significant differences after 4 days of infection for invasive growth in the tongue detected by histology using GMS staining (A), calcofluor white staining of C. albicans cells in tongue homogenates (B), the percent weight loss in the mice (C), or CFU per gram of tongue (D). The results represent averages from two to three independent infection experiments for each strain. Download FIG S2, PDF file, 1.4 MB. Copyright © 2019 Naseem et al. 2019 Naseem et al. This content is distributed under the terms of the Creative Commons Attribution 4.0 International license.

### Partial rescue of the *rvs*Δ invasion defect by raising the ambient CO_2_ level.

**FIG 6:**
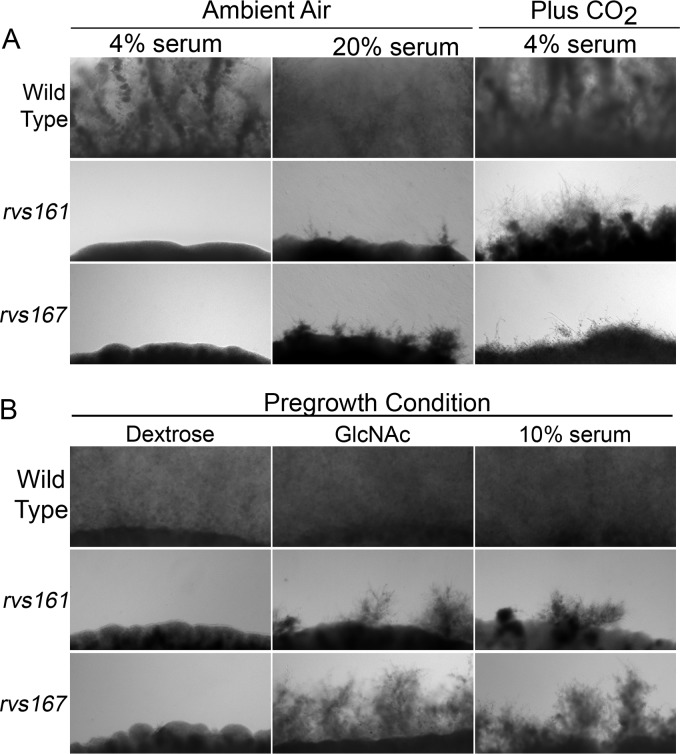
An enriched CO_2_ atmosphere stimulates invasive growth of the *rvs161*Δ and *rvs167*Δ mutants. Cells spotted onto agar plates were incubated for 3 days at 37°C, and the edge of the spot of cells was then photographed to record the extent of invasion. (A) Cells were spotted onto agar containing the indicated concentrations of bovine serum and then incubated in ambient air or in the presence of 5% CO_2_. (B) Cells were pregrown in YP medium with dextrose or with the hypha inducer 50 mM GlcNAc or YPD with 10% serum. The cultures were grown for 90 min at 37°C and then spotted onto agar plates containing 4% serum. Similar results were obtained in at least three independent experiments under each condition.

It was surprising that the *rvs*Δ mutants displayed such strong invasive-growth defects on the tongue given that our previous work showed that in the kidney, they formed long filamentous cells with minor morphological defects (39). This suggested that environmental differences between the oral cavity and the kidney influenced invasion. We therefore examined the effects of increasing ambient CO_2_ levels, since CO_2_ stimulates hyphal growth (18, 41, 42), and CO_2_ levels are expected to be different between the tongue and kidney. Interestingly, *rvs161*Δ cells grown in 5% CO_2_ formed hyphae that invaded agar (Fig. 6). Although the invasive hyphae were not as long as those seen for the wild type, this was nonetheless dramatically different from the lack of invasion seen for the *rvs161*Δ mutant grown under low-CO_2_ ambient conditions (∼0.04% CO_2_). The *rvs167*Δ mutant showed more limited invasion in the presence of CO_2_, but this was still improved relative to the low-CO_2_ conditions. Thus, the invasive-growth defect seen for the *rvs*Δ mutants is due in part to the differences in environmental conditions between the tongue and internal organs.

### Preinduction of hyphal morphology restores the ability of *rvs*Δ mutants to cause OPC.

Previous studies suggested that endocytosis could play an important role in hyphal initiation, as endocytosis is concentrated in the subapical region of the hyphal outgrowth and therefore has the potential to help focus morphogenesis at a narrow site of polarized growth (43, 44). To examine this, C. albicans cells were induced in liquid to form hyphae for 90 min and then added to agar plates containing 4% serum to monitor the extent of invasive growth. Interestingly, preinduction of hyphal growth with either GlcNAc or serum enabled the *rvs*Δ mutants to grow invasively in agar (Fig. 6). The invasive growth was not as extensive as that seen for the wild-type control cells. Nonetheless, it is very significant that invasive growth was readily detectable for preinduced *rvs*Δ mutant cells and not observed when the *rvs*Δ mutants were pregrown under noninducing conditions.

**FIG 7:**
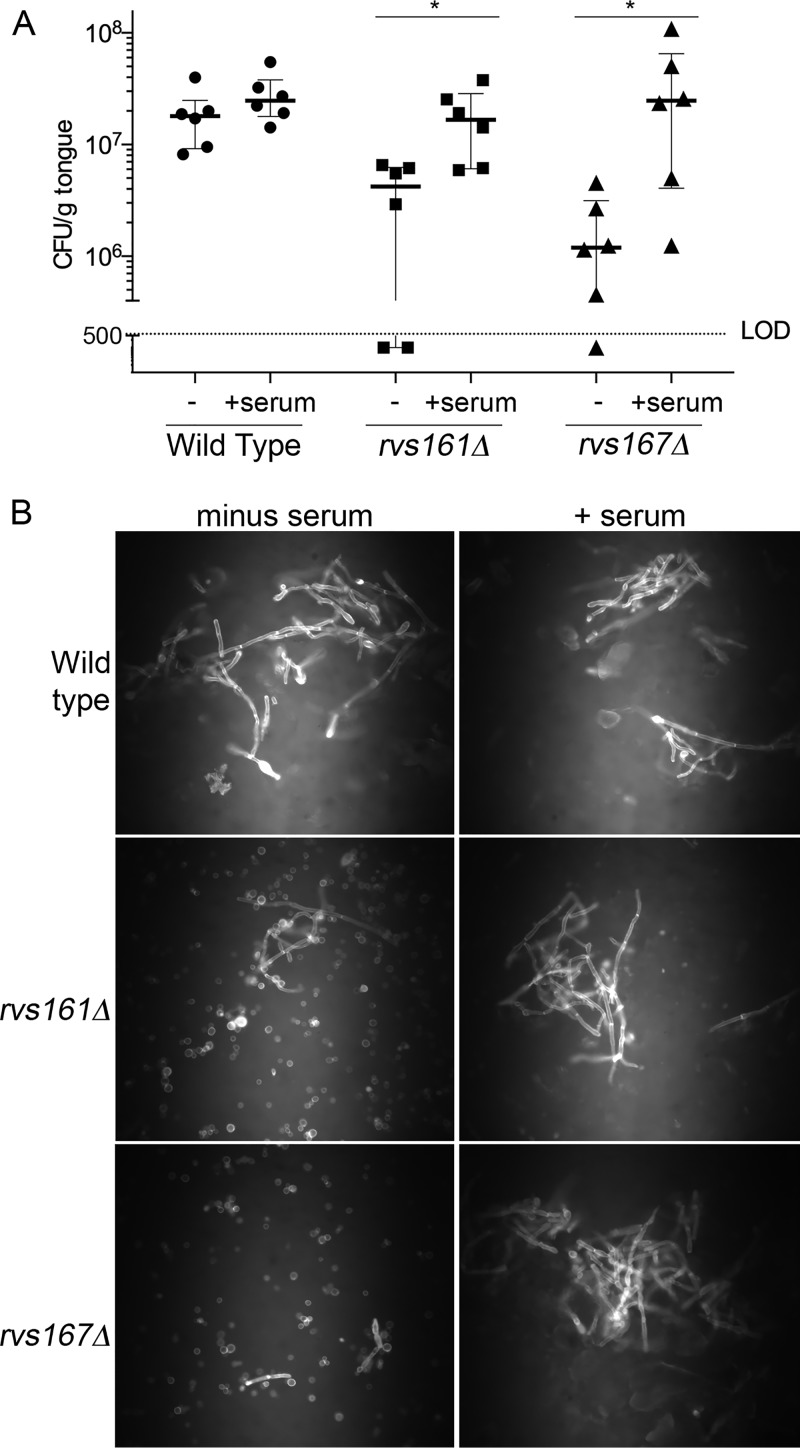
Induction of hyphal morphogenesis prior to oral infection rescues the OPC defect of the *rvs*Δ mutant strains. The strains were pregrown for 90 min in standard medium lacking serum (−) or in medium containing 10% bovine serum (+serum) to induce hyphal morphology. (A) The strains were then used to infect the oral cavity of mice, and 4 days later, the CFU per gram of tongue was determined. LOD indicates the limit of detection. Bars indicate medians and interquartile ranges. Statistical comparisons were made using a nonparametric Mann-Whitney test. (B) The remaining tongue homogenates were then treated with 1 M KOH to dissolve mouse tissue, and the C. albicans cell walls were then stained with the fluorescent dye calcofluor white and visualized by fluorescence microscopy. The results represent a summary of data from at least two independent infection experiments for each C. albicans strain.

We next determined whether preinduction of hyphal morphogenesis would improve the ability of the *rvs*Δ mutants to cause OPC. The *rvs*Δ cells were treated with serum for 90 min and then used to infect the oral cavity. A significant increase in CFU per gram of tongue was seen for preinduced cultures of both the *rvs161*Δ and *rvs167*Δ mutants relative to control cells that were not induced to form hyphae (Fig. 7). Examination of cell morphology by staining with calcofluor white indicated that a high proportion of the C. albicans cells in tongue homogenates from mice infected with preinduced *rvs*Δ cultures were growing in a filamentous manner, in contrast to the cells from mice infected with cells grown under standard conditions.

## DISCUSSION

### Strategies for identifying C. albicans mutants defective in OPC.

OPC is a common infection that causes severe morbidity (1), but it is much less studied in animal models than systemic candidiasis, in part because the time-consuming procedures used to initiate oral infections limit the number mice that can be tested (27). It is not practical to increase the throughput by testing pools of mutants because invasive strains will help noninvasive strains adhere in the oral cavity (9). Consequently, only about 24 mutants are listed as being defective in OPC in the *Candida* Genome Database (11, 17, 30–35, 45–52). To identify the mechanisms that promote invasive hyphal growth in the oral cavity, which is critical for C. albicans to cause OPC (1, 9), we first screened *in vitro* for mutants that that are defective in invading agar under strongly inducing conditions. Although many hyphal mutants have been identified previously, their impact has often been limited because they are defective only in responding to weak stimuli and can still invade a solid matrix in the presence of a strong hyphal inducer (e.g., serum). In this regard, it was interesting that the *rvs*Δ mutants, which had the strongest OPC defect, were the only mutants that we studied that still showed little or no invasive growth after a 1-week incubation (Table 2). This suggests that a longer incubation on agar plates could help future studies to select optimal mutants to test in OPC.

All nine mutants that we studied showed at least a partial defect in undergoing invasive hyphal growth in the oral mucosa, as evidenced by the presence of budding cells in histological analysis of the tongue that were not seen for the control strains. Since histology typically analyzes only a subset of the tongue, we confirmed the results by analyzing C. albicans morphology in a total tongue homogenate that had been treated with KOH to dissolve the mouse tissue (Fig. 4). The mutants identified in this study therefore represent an increase of about 35% in the number of strains known to have a defect in OPC (11, 17, 30–35, 45–52). These mutants are also among the first C. albicans strains to be reported to have clear defects in invasive hyphal growth in the oral mucosa of mice. Previously reported mutants that displayed lower fungal burdens in OPC were still hyphal, or in many cases, histology was not carried out, likely due to the expense (11, 17, 30–35, 45–52). Thus, calcofluor white staining of KOH-treated samples to detect fungal morphology represents a cost-effective alternative for future studies.

### Role of endocytosis in initiating C. albicans invasive growth.

A role for endocytosis was highlighted because the *rvs161*Δ and *rvs167*Δ mutants had the strongest OPC phenotype of the mutants that we tested. Rvs161 and Rvs167 are amphiphysin proteins needed for the scission phase of endocytosis (39). The *rvs*Δ mutants colonized the tongue at day 1 postinfection, similar to the wild-type control strain, indicating that there was no difference in initial adherence (Fig. 5). However, after 2 days of infection, the CFU per gram of tongue decreased for mice infected with the *rvs*Δ mutants relative to wild-type C. albicans, which correlated with the strong defect of the *rvs*Δ mutants in forming invasive hyphae (Fig. 3 and 4). An important role for endocytosis is also supported by reports that other membrane-trafficking mutants have defects in OPC, including *arf2*Δ, *arl1*Δ, *snf7*Δ, *vps15*Δ, *vps27*Δ, and *vps51*Δ mutants (35, 45, 46).

It was interesting that the *rvs*Δ mutants did not efficiently form hyphae in the oral cavity, since they grow as filamentous cells in liquid culture after stimulation with serum or GlcNAc and in the kidneys of mice during systemic infection (39, 53). The strong defect in oral versus systemic candidiasis suggested that the *rvs*Δ mutants were differentially affected by distinctive aspects of the oral environment (8, 54–56). The antimicrobial peptide histatin 5, which is found in saliva but not the kidney, is thought to play an important role in controlling C. albicans (57, 58). However, our previous studies found that the *rvs*Δ mutants were not more susceptible to histatin 5, as they were actually about 5-fold to 10-fold more resistant (39). Instead, our data indicate that a key difference between the oral cavity and other host sites is that the level of the hyphal inducer CO_2_ is expected to be high in internal organs, such as the kidney, but low in the oral cavity. This conclusion is supported by data showing that increasing the ambient levels of CO_2_ *in vitro* partially rescued the invasive-growth defects of the *rvs*Δ mutants (Fig. 6). This effect of CO_2_ appeared to be specific for the *rvs*Δ mutants, as CO_2_ did not have similar effects on the other mutants.

To better define the defects of the *rvs*Δ mutants, we examined their ability to grow invasively if they were first preinduced to form hyphae. Interestingly, preinduced *rvs*Δ cells were better able to grow invasively in agar and to cause OPC (Fig. 6 and 7). This indicates that the *rvs*Δ mutants have a stronger defect in initiation rather than maintenance of invasive hyphal growth. The analysis of preinduced strains is also significant, as it represents a new way to classify the defects of other OPC mutants. The endocytic defect of the C. albicans *rvs*Δ mutants (39, 59) suggests that proper membrane trafficking is important for directing the cell polarity machinery to promote hyphal morphogenesis. In particular, endocytosis has been proposed to play an important role in the initiation of hyphal growth. The active zone of endocytosis is subapical to the zone of polarized growth and is thought to enhance the efficiency of targeting growth to the hyphal tip by recycling lipids and limiting the spread of the cell wall-synthesizing enzymes below the active zone of growth at the hyphal tip (43, 44).

### Developing novel therapeutic approaches for OPC.

New therapeutic approaches are needed for treating OPC, as the number of antifungal drugs available is limited and their long-term prophylactic use by high-risk patients can lead to the emergence of drug resistant strains (60–62). The C. albicans mutants that we identified showed the potential of targeting invasive growth, as they all displayed at least partial defects in OPC. However, it seems likely that very strong impairment of hyphal growth will be required to have therapeutic value for treating OPC, since it appears that a subset of invasive hyphal cells can anchor other cells to the oral mucosa. Another important consideration is that the environmental influences that trigger invasive growth can differ between the oral cavity and internal organs, such as CO_2_ levels, which can lead to differential defects in oral versus systemic candidiasis (63). The ability of C. albicans to use distinct mechanisms to respond to different host environments should therefore be considered in developing new therapeutic strategies. Interestingly, recent studies showed that it was feasible to identify small molecules that inhibited endocytosis and hyphal growth (64) and that inhibitors of hyphal growth exhibit efficacy against OPC (65, 66). Furthermore, C. albicans endocytosis mutants, including *rvs*Δ strains, were more susceptible to killing by copper (67), suggesting that copper could enhance the therapeutic effects of blocking endocytosis. Thus, targeting hyphal growth is a promising therapeutic avenue that has the potential to impact both OPC and disseminated candidiasis (68).

## MATERIALS AND METHODS

### Strains and media.

The C. albicans strains used in these studies are described in Table S2 in the supplemental material. Cells were grown in rich YPD (yeast extract-peptone-dextrose) medium or a complete synthetic medium containing yeast nitrogen base, dextrose, amino acids, and uridine (69). Libraries of C. albicans mutant strains were obtained from the Fungal Genetics Stock Center (70).

### Agar invasion assays.

Libraries of deletion mutant strains that were constructed previously (21, 28, 29), along with strains in our own collection, were screened for the ability to grow invasively by spotting a sample of each strain onto 1.5% agar containing 4% fetal bovine serum. The edges of the spots were then monitored daily for the ability of the cells to form elongated hyphal filaments that grew invasively in the agar. Mutants that showed defects in invasive growth were then retested to confirm their mutant phenotype. PCR was used to confirm that the appropriate genes were deleted in the mutant strains. A selected set of mutants was then tested for invasive growth under a broader range of conditions, including 1% agar with serum, 4% agar with serum, as well as GlcNAc (yeast nitrogen base with 50 mM GlcNAc), alkaline pH 8 (150 mM pH 8 HEPES buffer), and spider medium (yeast nitrogen base, 1% mannitol, 1% nutrient broth, 0.2% K_2_HPO_4_ [pH 7.2] before autoclaving). Plates were incubated at 37°C. In some assays, cells were preinduced for 90 min in YPD plus 10% bovine serum or yeast extract-peptone plus 50 mM GlcNAc prior to application onto the surface of an agar plate.

### Oral infection assays.

Oral infections were carried out essentially as described previously (27). C. albicans strains were grown overnight at 30°C in YPD medium with 80 μg/ml uridine, reinoculated into fresh medium, and incubated again overnight at 30°C. Cells were harvested by centrifugation, washed twice in phosphate-buffered saline (PBS), counted in a hemocytometer, and then diluted to the appropriate density with PBS. To test the effects of preinduction of hyphae, cells were pregrown in YPD containing 10% bovine serum for 90 min at 37°C. C57BL/6 mice were injected 1 day before infection with cortisone acetate (catalog number C3130; Sigma-Aldrich) to induce immunosuppression. Cortisone acetate was dissolved in PBS with 0.05% (vol/vol) Tween 80 and administered at 225 mg/kg mouse in 0.2 ml. The mice were subsequently injected with additional doses of cortisone acetate 1 and 3 days after infection. Mice were sedated with 0.2 ml of ketamine (100 mg/kg of body weight) and xylazine (10 mg/kg). A calcium alginate swab (Puritan Medical Products Co., Guilford, ME) saturated with a suspension of 10^6^ C. albicans cells/ml was then placed under the tongue for 75 min. Mice were then monitored for weight loss or other signs of distress. Approximately 90% of the mice used were female, but similar results were obtained with male mice. In each set of experiments, selected mutant strains and a wild-type control strain of C. albicans were used to infect three mice each. Animal studies were conducted under guidelines established and approved by the Stony Brook University Institutional Animal Care and Use Committee (IACUC). Statistical analyses were carried out with GraphPad Prism software.

### CFU analysis.

Tongues were excised from mice at the indicated times after infection, weighed, placed in 5 ml PBS, and homogenized for 30 s with a tissue homogenizer (Pro Scientific, Inc., Oxford, CT). The CFU per gram of tongue tissue was then determined by plating serial dilutions of the homogenates onto YPD medium plates and incubating them at 30°C for 2 days.

### Calcofluor white staining.

Tongue homogenates were incubated in a solution of 10% KOH for 48 to 72 h to dissolve the mouse tissue. C. albicans cells were then harvested by centrifugation, and the cell walls were stained with calcofluor white (1 mg/ml), a fluorescent dye that binds to cell wall chitin. The cells were then examined by fluorescence microscopy to document cell morphology. In some cases, samples from several tongues were pooled to increase the number of cells that could be visualized by microscopy.

### Histology.

Tongues were fixed in 10% neutral buffered formalin, paraffin embedded, and sectioned at 5 μm. Sections were then stained with Grocott’s methenamine silver (GMS), which was performed by McClain Laboratories (Smithtown, NY). GMS stains fungal cells black, in contrast to the blue-green background of tongue cells.
