# Supplementary material for: Candida albicans rvs161Δ and rvs167Δ Endocytosis Mutants Are Defective in Invasion into the Oral Cavity
Source: mBio. 2019 Nov 12;10(6):e02503-19. doi: 10.1128/mBio.02503-19 (PMC6851284; doi:10.1128/mBio.02503-19)
Supplement: TABLE S2 [file mBio.02503-19-st002.pdf]

Supplementary Table S2. *C. albicans* strains used in this study

Strain	Short genotype	Full genotype
BWP17	parental strain	*his1::hisG/his1::hisG arg4::hisG/arg4::hisG ura3::λimm434/ ura3::λimm434*
DIC185	prototrophic WT control	*ura3::λimm434/URA3 his1::hisG/ HIS1 arg4::hisG/ ARG4*
YLD14-3	*rvs161Δ*	*rvs161Δ::ARG4/rvs161Δ::HIS1 URA3/ura3::λimm434 his1::hisG/his1::hisG arg4::hisG/arg4::hisG*
YLD16-11	*rvs167Δ*	*rvs167Δ::ARG4/rvs167Δ::HIS1 URA3/ura3::λimm434 his1::hisG/his1::hisG arg4::hisG/arg4::hisG*
	bni4Δ	*bni4Δ::URA3/ bni4Δ::URA3-ARG4-URA3 HIS1/his1::hisG arg4::hisG/arg4::hisG ura3::λimm434/ura3::λimm434*
	orf19.3751Δ	*orf19.3751Δ::URA3/ orf19.3751Δ::URA3-ARG4-URA3 HIS1/his1::hisG arg4::hisG/arg4::hisG ura3::λimm434/ura3::λimm434*
	spe1Δ	*spe1Δ::URA3/ spe1Δ::URA3-ARG4-URA3 HIS1/his1::hisG arg4::hisG/arg4::hisG ura3::λimm434/ura3::λimm434*
	orf19.716Δ	*orf19.716Δ::URA3/ orf19.716Δ::URA3-ARG4-URA3 HIS1/his1::hisG arg4::hisG/arg4::hisG ura3::λimm434/ura3::λimm434*
	orf19.2336Δ	*orf19.2336Δ::URA3/ orf19.2336Δ::URA3-ARG4-URA3 HIS1/his1::hisG arg4::hisG/arg4::hisG ura3::λimm434/ura3::λimm434*
	orf19.4880Δ	*orf19.4880Δ::URA3/ orf19.4880Δ::URA3-ARG4-URA3 HIS1/his1::hisG arg4::hisG/arg4::hisG ura3::λimm434/ura3::λimm434*

kre5Δ	*kre5Δ::HIS1/ kre5Δ::LEU2 his1Δ/his1Δ leu2Δ/leu2Δ ARG4/arg4Δ URA3/ura3::imm IRO1/iro1Δ::imm*
spf1Δ	*spf1Δ::HIS1/ spf1Δ::LEU2 his1Δ/his1Δ leu2Δ/leu2Δ ARG4/arg4Δ URA3/ura3::imm IRO1/iro1Δ::imm*
	spf1Δ::HIS1/ spf1Δ::LEU2 his1Δ/his1Δ leu2Δ/leu2Δ ARG4/arg4Δ URA3/ura3::imm IRO1/iro1Δ::imm
