# Supplementary material for: Candida albicans rvs161Δ and rvs167Δ Endocytosis Mutants Are Defective in Invasion into the Oral Cavity
Source: mBio. 2019 Nov 12;10(6):e02503-19. doi: 10.1128/mBio.02503-19 (PMC6851284; doi:10.1128/mBio.02503-19)
Supplement: FIG S1 [file mBio.02503-19-sf001.pdf]

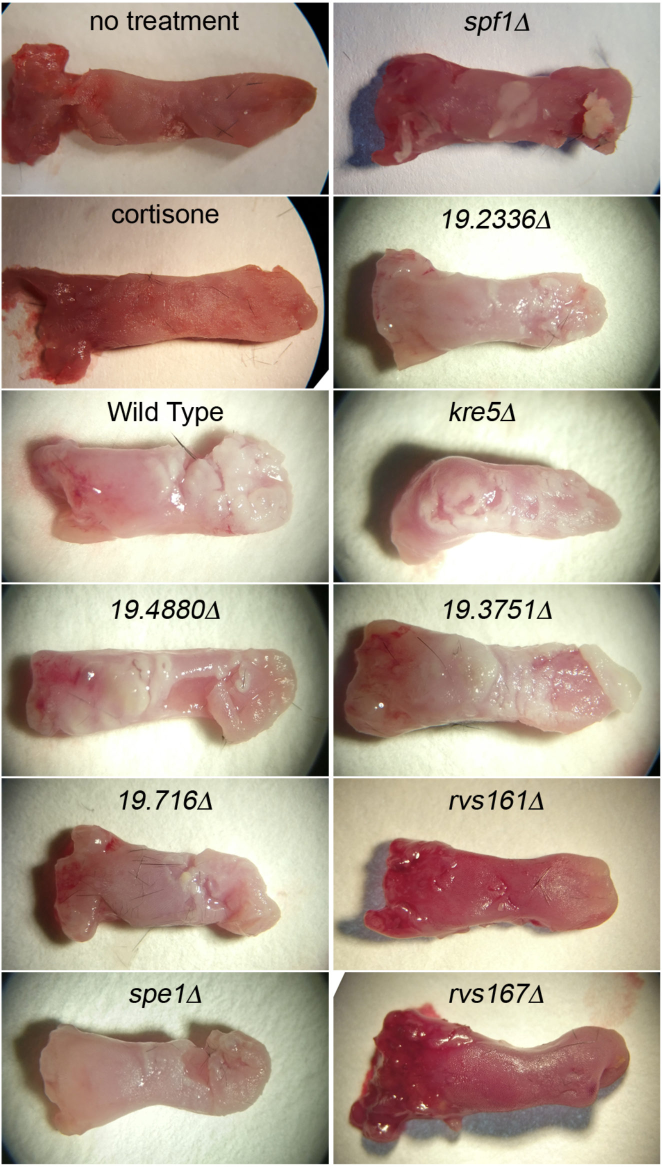
no treatment
spf1Δ
cortisone
19.2336Δ
Wild Type
kre5Δ
19.4880Δ
19.3751Δ
19.716Δ
rvs161Δ
spe1Δ
rvs167Δ
