# Supplementary figures and images for: Candida albicans rvs161Δ and rvs167Δ Endocytosis Mutants Are Defective in Invasion into the Oral Cavity
Source: mBio. 2019 Nov 12;10(6):e02503-19. doi: 10.1128/mBio.02503-19 (PMC6851284; doi:10.1128/mBio.02503-19)

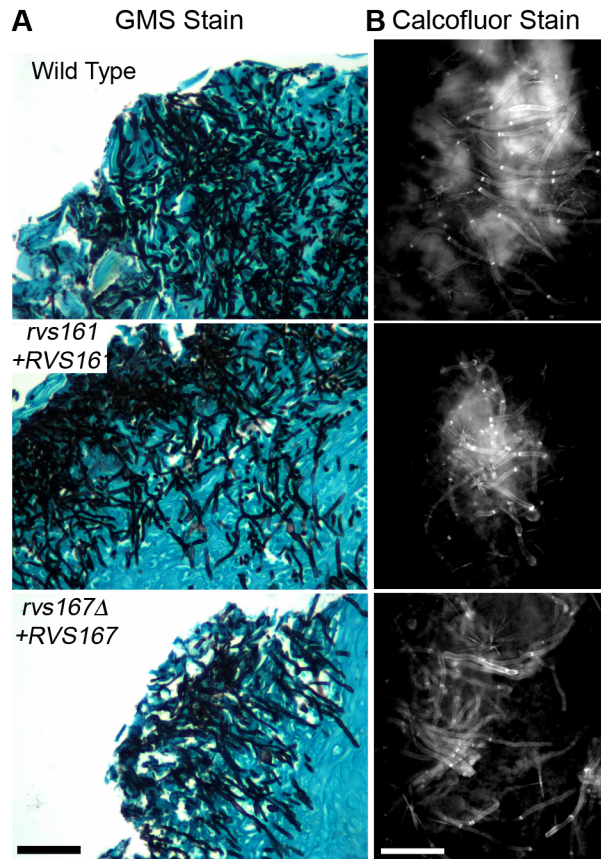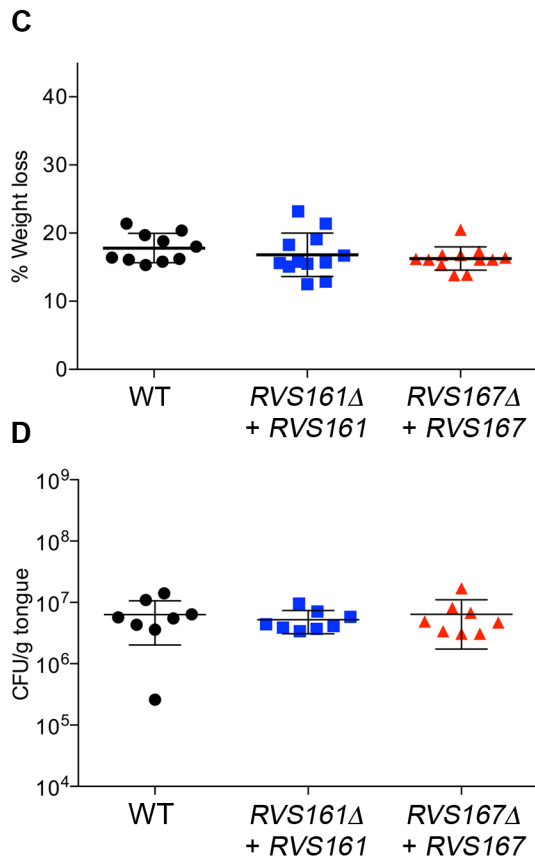

Supplement: FIG S2 [file mBio.02503-19-sf002.pdf]
